# Tempol effects on diabetic nephropathy in male rats

**DOI:** 10.15171/jrip.2016.16

**Published:** 2016-04-21

**Authors:** Akram Ranjbar, Hassan Ghasemi, Mahdi Hatami, Farahanaz Dadras, Tavakol Heidary Shayesteh, Farhad Khoshjou

**Affiliations:** ^1^Department of Toxicology and Pharmacology, School of Pharmacy, Hamadan University of Medical Sciences, Hamadan, Iran; ^2^Department of Biochemistry, Hamadan University of Medical Sciences, Hamadan, Iran; ^3^Department of Internal Medicine, Section of Nephrology, Iran University of Medical Sciences,Tehran, Iran; ^4^Urology and Nephrology Research Center, Hamadan University of Medical Sciences, Hamadan, Iran

**Keywords:** Tempol, Diabetic nephropathy, Oxidative stress, Superoxide dismutase, Glutathione peroxidase

## Abstract

**Introduction:** Diabetic nephropathy (DN) is the most common cause of the chronic kidney disease in the world. Oxidative stress on the other hand has a major and well known role in its pathophysiology.

**Objectives:** The aim of the study is to figure out if tempol, a synthetic antioxidant agent, modifies DN and to determine its relevance to changes of serum oxidative biomarkers.

**Materials and Methods:** Twenty-seven male rats were equally divided in to 4 groups (7 rats for each group). Group I (control or C), group II (diabetic or D), groups III (Tempol) which were given tempol (100 mg/kg/day) by gavages for 28 days and group IV (D&T) which includes diabetic rats that also received same dose of tempol. After treatment, blood samples were isolated. Enzymatic scavengers including catalase (CAT), glutathione peroxidase (GPx) and superoxide dismutase (SOD) activities, lipid peroxidation (LPO), total antioxidant capacity (TAC) and total thiol molecules (TTM) were measured. Blood urea nitrogen (BUN), creatinine (Cr) an albumin/Cr ratio were evaluated as well. Statistical differences were assessed with one-way analysis of variance (ANOVA) by SPSS followed by Tukey t test.

**Results:** Oxidative stress biomarkers modified and Alb/Cr ratio increased in diabetic group (II), however, they were altered to normal in group IV (D&T) compared with diabetic group (D).

**Conclusion:** Tempol can modify oxidative stress biomarkers and presumably nephropathy in diabetic rats.

**Implication for health policy/practice/research/medical education:**

Diabetic nephropathy (DN) is the major etiology of chronic kidney disease. Its pathophysiology has been widely studied. Oxidative stress has been known to be involved in it extensively. Thus a couple of antioxidative agent have been studied to figure out if they can modify DN and other consequences of diabetes mellitus (DM). In current study we tried to assess a couple of oxidative biomarkers in diabetic rats, following administration of tempol, a synthetic superoxide dismutase (SOD) mimetic, to show whether it can amend their changes and reduce proteinuria as well.

## Introduction

‎Diabetic nephropathy (DN) is the most important leading cause of end stage renal disease (1). Some 40% of patients with type 1 or 2 diabetes mellitus (DM) develop nephropathy after a latent period of about 15 years. It is characterized by proteinuria, reduced glomerular filtration rate (GFR), podocyte injury, mesangial matrix accumulation and tubulointerstitial damage (2). Oxidative stress has a major role in diabetes complications including DN (3). A growing amount of evidence indicates that the antioxidant capacity is decreased in diabetic patients (4). Animal and clinical studies have confirmed that antioxidant treatment plays an effective role in reducing demonstration of DN (5,6). glutathione peroxidase (GPx), catalase (CAT) and superoxide dismutase (SOD) and total antioxidant capacity (TAC) were significantly decreased in both type 1 and type 2 diabetic groups, with and without nephropathy, comparing with normal healthy individuals (7,8) and its decrement is coordinated to severity of microalbuminuria (9,10). SOD enzyme is upregulated in response to an increase in oxidative stress (11). This is a critical cellular defense mechanism. Thus reduction of SOD enzyme in the diabetic condition leads to renal cell injury. Exercised diabetic mice had significantly higher serum SOD compared with sedentary littermates(12). Several studies have investigated SOD activity and expression. Some, but not all of them, showed up-regulated renal SOD activity (13). Tempol, a SOD mimetic, is a well-known antioxidative agent and has been studied extensively in animal models. Tempol blocked the effect of glucose on rat glomerular mesangial cells to generate vascular endothelial growth factor (14). Cu/Zn SOD knockout mice developed more severe nephropathy following induction of DM which was reduced by oral tempol(15). Furthermore tempol administration to obese, hypertensive Zucker rats, reduced renal inflammation, proliferation and fibrosis but failed to reduce the proteinuria (16). Other studies, on the other side, showed reduction of proteinuria by tempol administration (17,18). In this study, we assessed tempol effect on serum CAT, SOD, GPx, TAC, total thiol molecules (TTM), lipid peroxidation (LPO) and proteinuria in different groups of rats including diabetic ones.

## Objectives

The aim of the study is to figure out if tempol, a synthetic antioxidant agent, modifies DN and to determine its relevance to changes of serum oxidative biomarkers.

## Materials and Methods

### Reagents and chemicals

Tetraethoxypropane (TEP), 2-thiobarbituric acid (TBA), trichloroacetic acid (TCA), n-butanol, ethylenediaminetetraacetic acid (EDTA), 2,4,6-tripyridyl-s-triazine (TPTZ) , GPx and SOD (Ransel kit, Randox Laboratories Ltd, Crumlin, UK), were used in this study. All other chemicals were obtained from the Sigma.

### Animals and treatments

Adult male Wistar rats weighing 180–250 g maintained on a 12-hour light/dark cycle with free access to tap water and standard laboratory chow were used. Animals were randomly divided into 4 groups of 7 animals and treated for 4 week by gavages. This experimental model of rats made diabetic with streptozotocin (STZ) injection has been validated in previous studies. Diabetes disease was induced by only a single intraperitoneal (i.p.) injection of STZ (60 mg/kg body weight) which was prepared by citrate buffer, pH 4.5 (19). The fasting blood glucose levels were determined 3 days after STZ injection by using a strip-operated blood glucose sensor. Animals were considered diabetic if plasma glucose levels exceeded 250 mg/dl. The groups were as follows: control group, tempol group (100 mg/kg/day), diabetic group and finally diabetic and tempol group. At the end of the treatment, 24 hours post the last dose of treatment, animals were killed, and urine and blood samples were collected in tubes and serum was isolated quickly and kept frozen at –80°C. All procedures for the treatment of animals were approved by the research ethics committee of the Hamadan University of Medical Sciences.

### Experimental Protocols

#### Kidney parameters

Blood urea nitrogen (BUN), creatinine (Cr) levels and urine albumin and Cr were estimated using an automated biochemistry machine ‎according to the standard procedure of kits.

#### Oxidative stress biomarkers

#### Measurement of Cu/Zn-SOD activity

The activity of Cu/Zn-SOD was measured using a commercial kit (Ransod kit, Randox

Laboratories Ltd, Crumlin, UK). Measurement of the enzyme was based on the generation of superoxide radicals produced by xanthine and xanthine oxidase and reacted with 2-(4-iodophenyl)-3-(4-nitrofenol) 5-phenyltetrazolium chloride (INT) to form a red formazan dye. The formazan was read at 505 nm. One unit of Cu/Zn-SOD was defined as the amount of enzyme necessary to produce 50% inhibition in the INT reduction rate.

#### Measurement of GPx activity

The amount of GPx was determined using a commercially available kit (Ransel kit, Randox

Laboratories Ltd, Crumlin, UK) by measuring the rate of oxidation of NADPH at 340 nm. A unit of enzyme was expressed as the amount of enzyme needed to oxidize 1 nmol of NADPH oxidase/minute.

#### Measurement of lipid peroxidation

The LPO product in tissues was determined by TBA reagent expressed as the extent of malondialdehyde (MDA) productions during an acid heating reaction. The calibration curve of tetraethoxypropane standard solutions was used to determine the concentrations of TBA+MDA adducts in samples (20).

#### Assay of total antioxidant capacity

It was measured by ferric reducing ability of plasma (FRAP) method. This method is based on the ability of plasma in reducing Fe^3+^ to Fe^2+^ in the presence of TPTZ. The reaction of Fe^2+^ and TPTZ gives a complex with blue color and maximum absorbance in 593 nm (21).

#### Assay of total thiol molecules

To evaluate the plasma TTM, DTNB was used as a reagent. DTNB reacts with thiol molecules and create a yellow complex which has good absorbance at 412 nm in spectrophotometer (22).

#### Ethical issues

The research followed the tenets of the Declaration of Helsinki. The research was approved by ethical committee of Hamedan University of Medical Sciences. Prior to the experiment, the protocols were confirmed to be in accordance with the Guidelines of Animal Ethics Committee of Hamedan University of Medical Sciences.

#### Statistical analysis

Mean and standard error values were determined for all the parameters and the results were expressed as mean ± SEM. All data were analyzed with SPSS version 16 employing one-way analysis of variance (ANOVA) followed by Tukey post hoc test. Differences between groups was considered significant when *P*<0.05.

## Results

After 3 days of follow up, diabetes was induced in rats which were injected STZ intraperitoneally. DN also confirmed by significant rising of albumin/Cr ratio (ACR) in diabetic (group D) rats after 28 days follow up (#8 folds). Serum urea, but not serum Cr, increased in group D significantly (Table 1).

**Table 1:** BUN, Cr and Alb/Cr in different groups of rats

**Groups**	**BUN (mg/dl)**	**Cr (mg/dl)**	**Alb/Cr**
Control	44 ± 3.39	0.61 ± 0.06	258 ± 28
Diabetic	60.4 ± 2.8^a^	0.66 ± 0.04	2000 ± 28^a^
Tempol	49.8 ± 4.04	0.59 ± 0.04	932 ± 17
Diabetic±Tempol	51.7 ± 3.92	0.67 ± 0.05	297 ± 14^b^

Abbreviations: Cr, creatinine; Alb/Cr, albumin/creatinine ratio; BUN, blood urea nitrogen. ^a^Significantly different from control group at *P* < 0.05; ^b^Significantly different from fiabetic group at *P* < 0.05.

Serum samples of different groups also were collected after 4 weeks of treatment and some oxidative stress indexes including TAC were measured.

### Glutathione peroxidase

GPx showed significant rise in group D, however decreased significantly in group D&T compared to group D (Table 2).

**Table 2:** Antioxidative enzymes amounts in different groups of rats

**Groups**	**TAC (µmol/ml)Mean ±SE**	**GPx (U/l)Mean ± SE**	**CAT (U/ml)Mean ± SE**	**SOD (U/ml)Mean ± SE**	**LPO (nmol/ml)Mean ± SE**	**TTM (nmol/ml)Mean ±SE**
Control	3.2± 0.8	57.9 ± 5.3	17.3 ± 5.1	0.32 ± 0.08	1 ± 0.19	0.29 ± 0.04
Diabetic	1.7 ± 0.26	93.2 ± 4.6^a^	52.7 ± 8.6^a^	0.62 ± 0.13^a^	1.9 ± 0.23^a^	0.15 ± 0.06
Tempol	5.9 ± 0.36^b^	46.2 ± 4.6^b^	12.9 ± 4.00^b^	0.22 ± 0.05^b^	0.83 ± 0.22^b^	0.59 ± 0.04^b^
Tempol & diabetic	3.8 ± 0.39^b^	50.03 ± 7.2^b^	31.03 ± 7.5	0.36 ± 0.09	1.68 ± 0.24	0.26 ± 0.06

Abbreviation: GPx, glutathione peroxidase; CAT, catalase; SOD, superoxide dismutase; TAC, total antioxidant capacity; total TTM, thiol molecules; LPO, lipid peroxidation; SE, standard error. ^a^Significantly different from control group at *P* < 0.05; ^b^Significantly different from fiabetic group at *P* < 0.05.

### Catalase, superoxide dismutase and lipid peroxidation

Other oxidative stress indexes i.e. CAT, SOD and LPO showed similar results. All of them raised in group D significantly and also decreased in group D&T compared to last group (Table 2).

### Total antioxidant capacity and Total thiol Molecules

TAC and TTM decreased in group D compared to group C. On the other hand, they increased in D&T compared to group D (significantly for TAC) (Table 2). In group T, all of oxidative biomarkers were significantly different with group D.

## Discussion

Oxidative stress has a well-known role in pathophysiology of DN, which can be assessed by its biomarkers. On the other hand, it has been shown that in STZ induced diabetic rats, plasma glucose level was not affected by tempol treatment (23,24). Hence its protective effects in DN originates from antioxidant properties rather than reducing plasma glucose. SOD is an antioxidant enzyme present in the various cellular organelles including inner mitochondrial space. Its primary function is to lower intracellular concentrations of superoxide anion (25). Multiple variations in cytosolic Zn-Cu SOD (SOD1) are significantly associated with severe nephropathy (26). In present study serum SOD and CAT showed significant rise in group D, and decreased in group D&T compared to group D. Arellano-Buendía et al in a study (27) showed the similar results, although other studies demonstrated decrease of these biomarkers of oxidative stress. Different results can be due to various duration of the studies (28 days versus 2 to 3 months). Study of different mice showed higher values of SOD activity in diabetic than nondiabetic ones (at 5 weeks of age) as well (28). This finding suggests that initial response of renal SOD enzyme to hyperglycemia is amplification. Thus, reduction of SOD enzyme in mouse kidney is caused by the secondary factor of chronic hyperglycemia (29,30). On the other hand, severity of albuminuria had a negative correlation with plasma GPx concentrations (31,32). Furthermore, rats with STZ-induced diabetes had lower levels of GPx and TAC in the kidney than that in the control rats (33,34). In present study, GPx and LPO increased in diabetic group significantly and also decreased in diabetic group which treated by tempol (D&T group). TAC and TTM also, decreased in diabetic group compared to controls. However, they increased in group D&T compared to group D. According to different studies, antioxidant enzymes levels changes in DN. In our study SOD, GPx and LPO increased. It is in line with some, but not all of other investigations. It is theorized that in short term these enzymes increase, however chronic hyperglycemia and inflammation cause decline of them. Furthermore TAC and TTG reduced.

## Conclusion

During first month following induction of diabetes in rat, oxidative stress biomarkers (i.e. SOD, GPx, CAT and LPO) increased. All of aforementioned changes besides surge of proteinuria are modified by tempol administration. Thus it is implied that tempol can ameliorate diabetic kidney disease.

## Limitations of the study

We did not measure and compare GFR in different groups of rats.

## Authors’ contribution

AR designed the study. HG, MH and THS collected the data. FD contributed to the writing process. FK was the study supervisor, contributed to all aspect of the study and provided the final manuscript. All authors read and approved the paper.

## Conflicts of interest

The authors declare that they have no conflicting interest.

## Ethical considerations

Ethical issues (including plagiarism, data fabrication, double publication) have been completely observed by the authors.

## Funding/Support

This research project was approved and supported by research Deputy of Hamadan University of medical sciences (Grant No. 9211013647).
